# Real-world progression-free survival of CDK4/6 inhibitors plus an aromatase inhibitor in HR-positive/HER2-negative metastatic breast cancer in United States routine clinical practice

**DOI:** 10.1016/j.esmoop.2025.105570

**Published:** 2025-09-01

**Authors:** H.S. Rugo, R.M. Layman, F. Lynce, X. Liu, B. Li, L. McRoy, A.B. Cohen, M. Estevez, G. Curigliano, A. Brufsky

**Affiliations:** 1 Department of Medical Oncology and Therapeutics Research, City of Hope Comprehensive Cancer Center, Duarte; 2 The University of Texas MD Anderson Cancer Center, Houston, USA; 3 Dana-Farber Cancer Institute, Boston, USA; 4 Pfizer Inc., New York, USA; 5 Flatiron Health Inc., New York, USA; 6 NYU Langone School of Medicine, New York, USA; 7 European Institute of Oncology, IRCCS, Milan; 8 Department of Oncology and Hemato-Oncology, University of Milano, Milan, Italy; 9 UPMC Hillman Cancer Center, University of Pittsburgh Medical Center, Pittsburgh, USA

**Keywords:** comparative effectiveness, cyclin-dependent kinase 4/6 inhibitor, abemaciclib, ribociclib, palbociclib

## Abstract

**Background:**

All three cyclin-dependent kinase 4/6 inhibitors (CDK4/6i; palbociclib, ribociclib, and abemaciclib) plus aromatase inhibitor (AI) significantly prolonged progression-free survival (PFS) versus placebo plus AI and achieved a similar reduction in risk of disease progression in randomized controlled trials (RCTs) evaluating first-line (1L) treatment of hormone receptor (HR)-positive/human epidermal growth factor receptor 2 (HER2)-negative metastatic breast cancer (mBC). To date, there have been no head-to-head RCT data comparing CDK4/6i, and most real-world comparative effectiveness studies were limited by small sample sizes and/or short follow-up. In this analysis, we compared real-world PFS (rwPFS) in patients with HR-positive/HER2-negative mBC receiving 1L CDK4/6i plus AI in United States routine clinical practice.

**Patients and methods:**

P-VERIFY was a retrospective comparative effectiveness study using a US nationwide deidentified electronic health record-derived longitudinal database. Patients had HR-positive/HER2-negative mBC, were ≥18 years of age, and started 1L CDK4/6i plus AI between February 2015 and November 2023. rwPFS was defined as months from CDK4/6i plus AI initiation to disease progression or death from any cause. Stabilized inverse probability of treatment weighting (sIPTW) as primary analysis was used to balance baseline characteristics. Multivariable Cox proportional hazards model was carried out as a sensitivity analysis.

**Results:**

Of 9146 eligible patients, 6831, 1279, and 1036 received palbociclib, ribociclib, and abemaciclib, respectively, plus AI. Baseline characteristics were generally balanced between treatment groups after sIPTW. Median [95% confidence interval (CI)] rwPFS after sIPTW was 22.7 (21.6-23.8), 22.9 (21.0-25.6), and 22.9 (20.2-26.5) months in the palbociclib, ribociclib, and abemaciclib groups, respectively. After sIPTW, there were no significant rwPFS differences (all *P* > 0.05) between ribociclib versus palbociclib (adjusted hazard ratio 0.97, 95% CI 0.88-1.07), abemaciclib versus palbociclib (0.96, 0.86-1.06), and abemaciclib versus ribociclib (0.98, 0.86-1.12). Findings were generally consistent across subgroups and sensitivity analyses.

**Conclusions:**

Our study, the largest real-world CDK4/6i comparative effectiveness study to date, demonstrated no significant rwPFS differences between 1L palbociclib, ribociclib, and abemaciclib, plus AI, in patients with HR-positive/HER2-negative mBC.

## Highlights

• P-VERIFY is the largest real-world study to date comparing rwPFS between CDK4/6i plus AI for HR-positive/HER2-negative mBC.
• There were no significant rwPFS differences between 1L treatment with palbociclib, ribociclib, or abemaciclib, plus an AI.
• Findings were generally consistent in subgroup and sensitivity analyses.

## Graphical abstract

**Figure:**
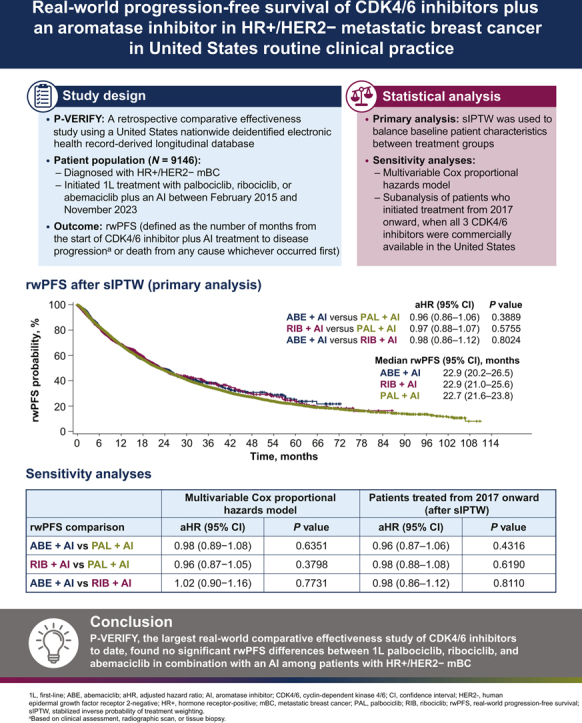

## Introduction

The majority of patients with breast cancer have hormone receptor (HR)-positive/human epidermal growth factor receptor 2 (HER2)-negative disease, and nearly 30% of patients initially diagnosed with early-stage breast cancer will ultimately develop metastatic breast cancer (mBC).^1^^,^^2^ Treatment with a cyclin-dependent kinase 4/6 (CDK4/6) inhibitor combined with endocrine therapy (ET) has emerged as the preferred first-line (1L) regimen for patients with HR-positive/HER2-negative advanced/mBC.^3^^,^^4^ There are currently three CDK4/6 inhibitors that have been approved by the United States Food and Drug Administration for treatment of HR-positive/HER2-negative mBC; palbociclib was the first to gain approval in 2015, followed by ribociclib and abemaciclib in 2017.^5^, ^6^, ^7^ In registrational phase III randomized controlled trials (RCTs) in patients with HR-positive/HER2-negative mBC, 1L treatment with a CDK4/6 inhibitor (palbociclib in PALOMA-2; ribociclib in MONALEESA-2; abemaciclib in MONARCH-3) plus ET demonstrated a significant prolongation of progression-free survival (PFS; primary endpoint) when compared with control treatment (placebo plus ET).^8^, ^9^, ^10^ A similar reduction in the risk of disease progression was observed with CDK4/6 inhibitor plus ET versus control treatment across these RCTs, with hazard ratios (HRs) ranging from 0.54 to 0.57 (all *P* < 0.0001). Across all three RCTs, median overall survival (OS; secondary endpoint) was numerically prolonged in the CDK4/6 inhibitor plus ET arm versus the control arm, although only ribociclib demonstrated a statistically significant prolongation of OS.^11^, ^12^, ^13^

To date, there have been no head-to-head RCT data comparing the efficacy of different CDK4/6 inhibitors in treating patients with HR-positive/HER2-negative mBC. In the absence of head-to-head RCTs, real-world evidence generated from real-world data is increasingly valued as a complement to RCT data.^14^ A growing body of evidence has evaluated the relative effectiveness of CDK4/6 inhibitors in patients with HR-positive/HER2-negative mBC in the real-world setting.^15^, ^16^, ^17^, ^18^, ^19^, ^20^, ^21^, ^22^, ^23^, ^24^, ^25^, ^26^ Of the real-world studies evaluating the relative risk of disease progression, most did not show significant differences in real-world PFS (rwPFS) between CDK4/6 inhibitors when used in combination with ET,^15^, ^16^, ^17^^,^^20^^,^^22^^,^^24^, ^25^, ^26^ with a couple of exceptions.^19^^,^^23^ However, prior studies were limited by small sample sizes and/or short follow-up, and definitions of rwPFS varied across studies. In this context, real-world studies with larger sample sizes and longer follow-up are needed to further investigate comparative rwPFS in patients receiving different CDK4/6 inhibitors in combination with ET.

To our knowledge, the Palbociclib Verifying Evidence of Real-world Impact Study (P-VERIFY) is the largest real-world study ever conducted to compare the effectiveness of the three CDK4/6 inhibitors. P-VERIFY used real-world data from a nationwide deidentified database derived from electronic health records of patients treated in routine clinical practice in the United States (US). Previously reported results from P-VERIFY showed no significant OS differences [all adjusted HRs (aHRs) 0.94-1.00, *P* > 0.05] in pairwise comparisons of CDK4/6 inhibitors (palbociclib, ribociclib, and abemaciclib) when used in combination with an aromatase inhibitor (AI) as 1L treatment for patients with HR-positive/HER2-negative mBC.^27^ The current P-VERIFY study aimed to compare rwPFS of patients with HR-positive/HER2-negative mBC receiving 1L palbociclib, ribociclib, or abemaciclib, in combination with an AI, in routine clinical practice.

## Patients and methods

### Study design

P-VERIFY (NCT06495164) was a retrospective comparative effectiveness study, for which detailed methods have been described previously.^27^ P-VERIFY used the Flatiron Health Research Database, a United States nationwide electronic health record-derived database that included deidentified data from >750 000 patients with breast cancer at the time of the study. This database contained structured and unstructured patient-level data, which were curated using machine learning-enabled natural language processing and technology-enabled abstraction.^28^, ^29^, ^30^ Flatiron Health’s quality and performance assessment frameworks were used to validate study variables.^31^, ^32^, ^33^

Patients included in this analysis had HR-positive/HER2-negative mBC; were ≥18 years of age at mBC diagnosis; started index treatment (palbociclib, ribociclib, or abemaciclib in combination with an AI) as 1L therapy up to 14 days before or 90 days after mBC diagnosis between February 2015 and November 2023; and had ≥6 months of potential follow-up time from the start of index treatment until the data cut-off date (31 May 2024). Patients were excluded if they participated in a clinical trial during the study period. Patients were assessed from the start of index treatment until the data cut-off date, death, or last medical activity, whichever occurred first. A subanalysis of patients who initiated treatment from 2017 onward, when all three CDK4/6 inhibitors were commercially available in the United States, was also carried out.

This study was conducted in accordance with the Guidelines for Good Pharmacoepidemiology Practices issued by the International Society for Pharmacoepidemiology (ISPE), Good Practices for Outcomes Research issued by the International Society for Pharmacoeconomics and Outcomes Research (ISPOR), and Good Practices for Real-World Data Studies of Treatment and/or Comparative Effectiveness issued jointly by ISPOR and ISPE. As a retrospective analysis of a deidentified database, this study was exempt from institutional review board approval and did not require informed consent.

### Outcome

rwPFS was defined as the number of months from the start of CDK4/6 inhibitor plus AI treatment to death from any cause or disease progression (based on clinical assessment, radiographic scan, or tissue biopsy), whichever occurred first.^34^, ^35^, ^36^ Patients who were alive and did not have disease progression were censored at the date of initiation of the next line of therapy, the date of their last medical activity, or the data cut-off date, whichever came first.

### Statistical analysis

Descriptive statistics were used for baseline demographic and clinical characteristics. rwPFS was summarized using the Kaplan–Meier method and displayed graphically. Cox proportional hazards models were used to compute HRs and their corresponding 95% confidence intervals (CIs). rwPFS was compared both before (unadjusted analysis) and after stabilized inverse probability of treatment weighting (sIPTW; primary analysis), which was implemented to balance baseline demographic and clinical characteristics between treatment groups. The sIPTW method used propensity scores, which were estimated via a multivariable multinomial logistic regression model that incorporated the following variables: age, sex, race, practice type, Eastern Cooperative Oncology Group performance status, stage of disease at initial diagnosis, visceral metastasis, bone-only metastasis, number of disease sites, and disease-free interval (the time from initial diagnosis of breast cancer to diagnosis of mBC). These same variables were included as covariates in a multivariable Cox proportional hazards model, which was used as a sensitivity analysis. All statistical analyses were carried out using SAS version 9.4 (SAS Institute Inc, Cary, NC).

## Results

### Patients

A total of 9146 patients with HR-positive/HER2-negative mBC who initiated treatment with palbociclib plus AI (*n* = 6831), ribociclib plus AI (*n* = 1279), or abemaciclib plus AI (*n* = 1036) between February 2015 and November 2023 were eligible for this analysis (Supplementary Figure S1, available at https://doi.org/10.1016/j.esmoop.2025.105570). Of the 9146 patients, 8161 (89.2%) started both CDK4/6 inhibitor and an AI within 28 days. The proportions of patients starting both CDK4/6 inhibitor and an AI within 28 days were very similar among the three CDK4/6 inhibitor groups: 89.0% in palbociclib plus AI, 91.7% in ribociclib plus AI, and 87.5% in abemaciclib plus AI. Baseline patient characteristics before and after sIPTW are presented in Supplementary Table S1, available at https://doi.org/10.1016/j.esmoop.2025.105570. Median age at mBC diagnosis was highest in the palbociclib group (66 versus 64 years in both the ribociclib and abemaciclib groups), whereas the ribociclib group had the highest proportion of premenopausal patients (28.5% versus 17.0% and 22.8% in the palbociclib and abemaciclib groups, respectively). The abemaciclib group had more patients with visceral metastases (40.7%) than the palbociclib and ribociclib groups (33.9% and 34.4%, respectively), and fewer patients with bone-only metastasis (40.6% versus 47.2% and 47.1%). After sIPTW, baseline demographics and clinical characteristics were generally balanced between treatment groups. In each treatment group, arithmetic median duration of follow-up remained consistent before and after sIPTW, at 33 months in the palbociclib group, ∼16 months in the ribociclib group, and ∼21 months in the abemaciclib group.

### Real-world progression-free survival

**Figure 1:**
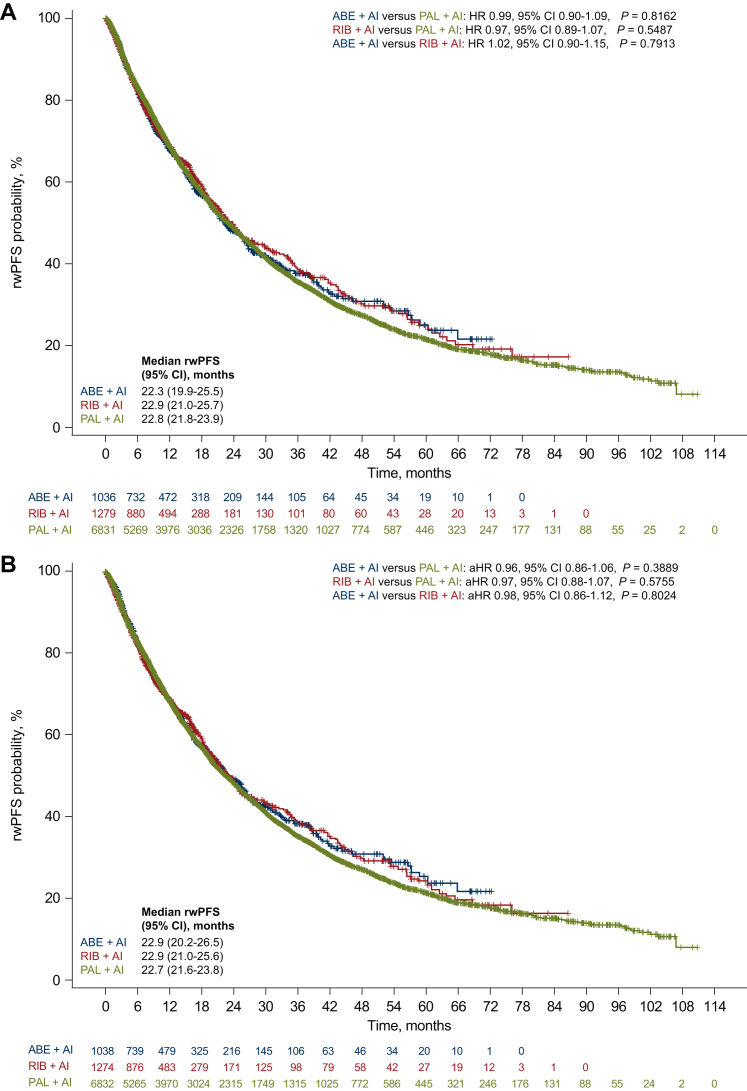
**Kaplan-Meier plot of rwPFS before and after sIPTW.** rwPFS in the (A) unadjusted analysis and (B) after sIPTW among the three CDK4/6 inhibitors.ABE, abemaciclib; AI, aromatase inhibitor; CDK4/6, cyclin-dependent kinase 4/6; CI, confidence interval; PAL, palbociclib; rwPFS, real-world progression-free survival; RIB, ribociclib; sIPTW, stabilized inverse probability of treatment weighting.

**Figure 2:**
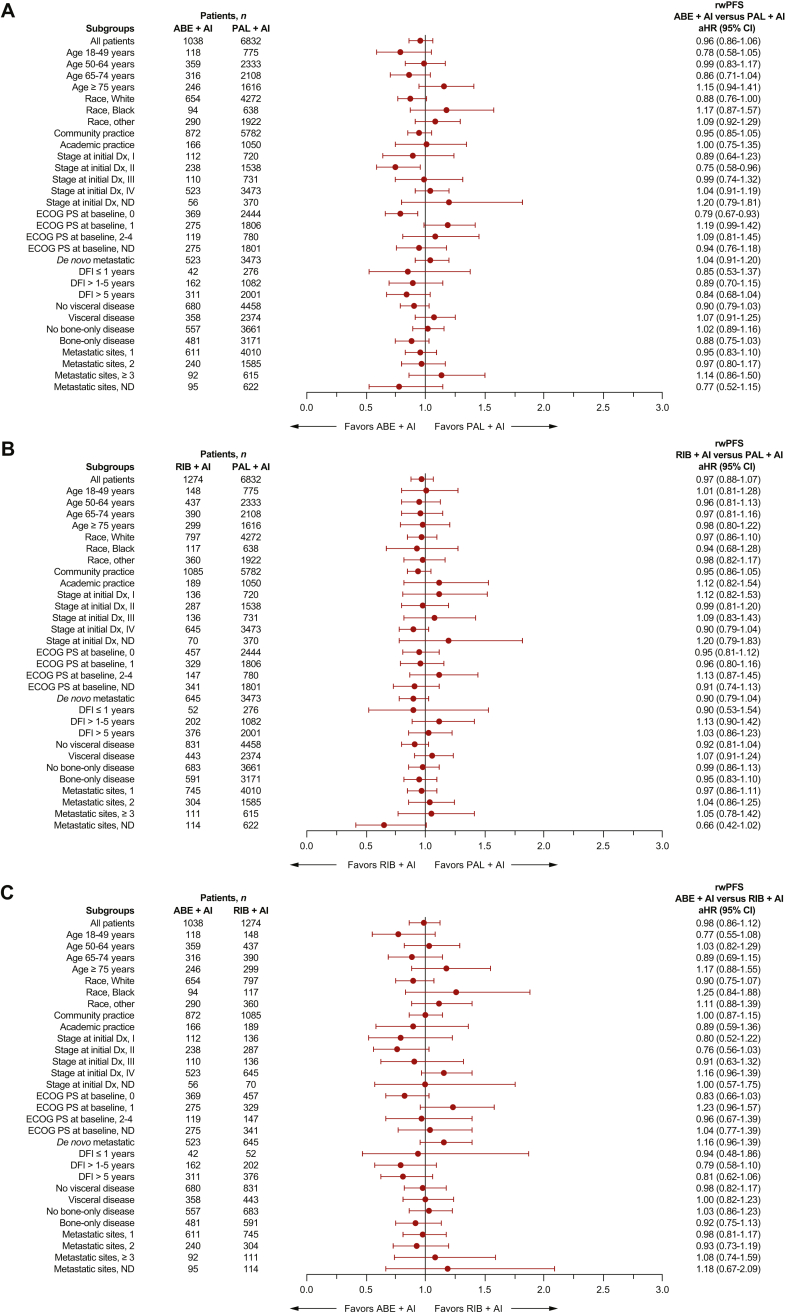
**Forest plot of rwPFS by subgroup after sIPTW.** (A) Abemaciclib + AI versus palbociclib + AI, (B) ribociclib + AI versus palbociclib + AI, and (C) abemaciclib + AI versus ribociclib + AI. ABE, abemaciclib; AI, aromatase inhibitor; DFI, disease-free interval; Dx, diagnosis; ECOG PS, Eastern Cooperative Oncology Group performance status; ND, not determined; PAL, palbociclib; RIB, ribociclib; rwPFS, real-world progression-free survival; sIPTW, stabilized inverse probability of treatment weighting.

Median rwPFS before and after sIPTW was 22.8 (95% CI 21.8-23.9) and 22.7 (95% CI 21.6-23.8) months, respectively, for palbociclib plus AI; 22.9 (95% CI 21.0-25.7) and 22.9 (95% CI 21.0-25.6) months for ribociclib plus AI; and 22.3 (95% CI 19.9-25.5) and 22.9 (95% CI 20.2-26.5) months for abemaciclib plus AI (Figure 1A and B). In the unadjusted analysis, there were no significant differences in rwPFS when comparing the ribociclib and palbociclib groups (HR 0.97, 95% CI 0.89-1.07, *P* = 0.5487), the abemaciclib and palbociclib groups (HR 0.99, 95% CI 0.90-1.09, *P* = 0.8162), and the abemaciclib and ribociclib groups (HR 1.02, 95% CI 0.90-1.15, *P* = 0.7913; Figure 1A). Similarly, after sIPTW, there were no significant rwPFS differences for each of these pairwise group comparisons, with an aHR of 0.97 (95% CI 0.88-1.07, *P* = 0.5755), 0.96 (95% CI 0.86-1.06, *P* = 0.3889), and 0.98 (95% CI 0.86-1.12, *P* = 0.8024), respectively (Figure 1B). Results from the subgroup analysis of rwPFS after sIPTW were generally consistent with those from the overall cohort for each treatment group comparison (Figure 2).

A multivariable Cox proportional hazards model as a sensitivity analysis also showed no significant differences in rwPFS between treatment groups. The aHR for each pairwise group comparison was 0.96 (95% CI 0.87-1.05, *P* = 0.3798) for ribociclib versus palbociclib, 0.98 (95% CI 0.89-1.08, *P* = 0.6351) for abemaciclib versus palbociclib, and 1.02 (95% CI 0.90-1.16, *P* = 0.7731) for abemaciclib versus ribociclib.

### Subanalysis of patients who started index treatment from 2017 onward

We carried out a subanalysis to focus on patients who started treatment after all three CDK4/6 inhibitors were approved and available for use in the United States. This subanalysis included 5735, 1279, and 1036 patients in the palbociclib, ribociclib, and abemaciclib groups, respectively. Baseline demographics and clinical characteristics before and after sIPTW for this subset of patients are presented in Supplementary Table S2, available at https://doi.org/10.1016/j.esmoop.2025.105570. After sIPTW, patient characteristics were generally balanced between treatment groups.

**Figure 3:**
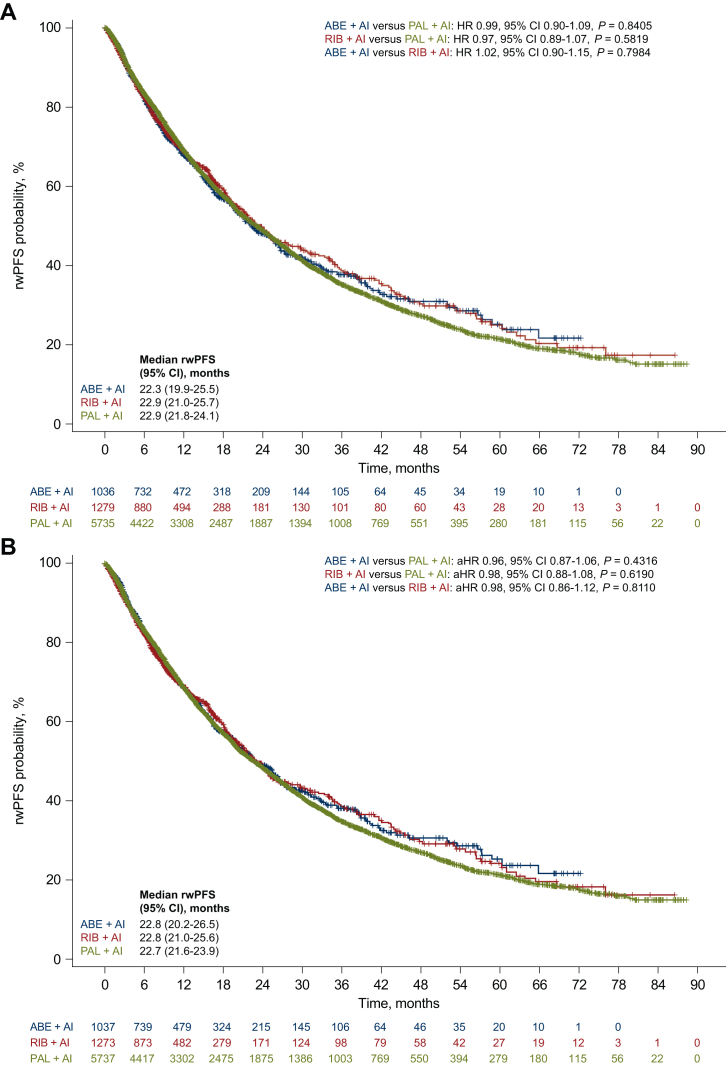
**Kaplan-Meier plot of rwPFS before and after sIPTW.** rwPFS in the (A) unadjusted analysis and (B) after sIPTW among the three CDK4/6 inhibitors.ABE, abemaciclib; AI, aromatase inhibitor; CDK4/6, cyclin-dependent kinase 4/6; CI, confidence interval; PAL, palbociclib; rwPFS, real-world progression-free survival; RIB, ribociclib; sIPTW, stabilized inverse probability of treatment weighting.

**Figure 4:**
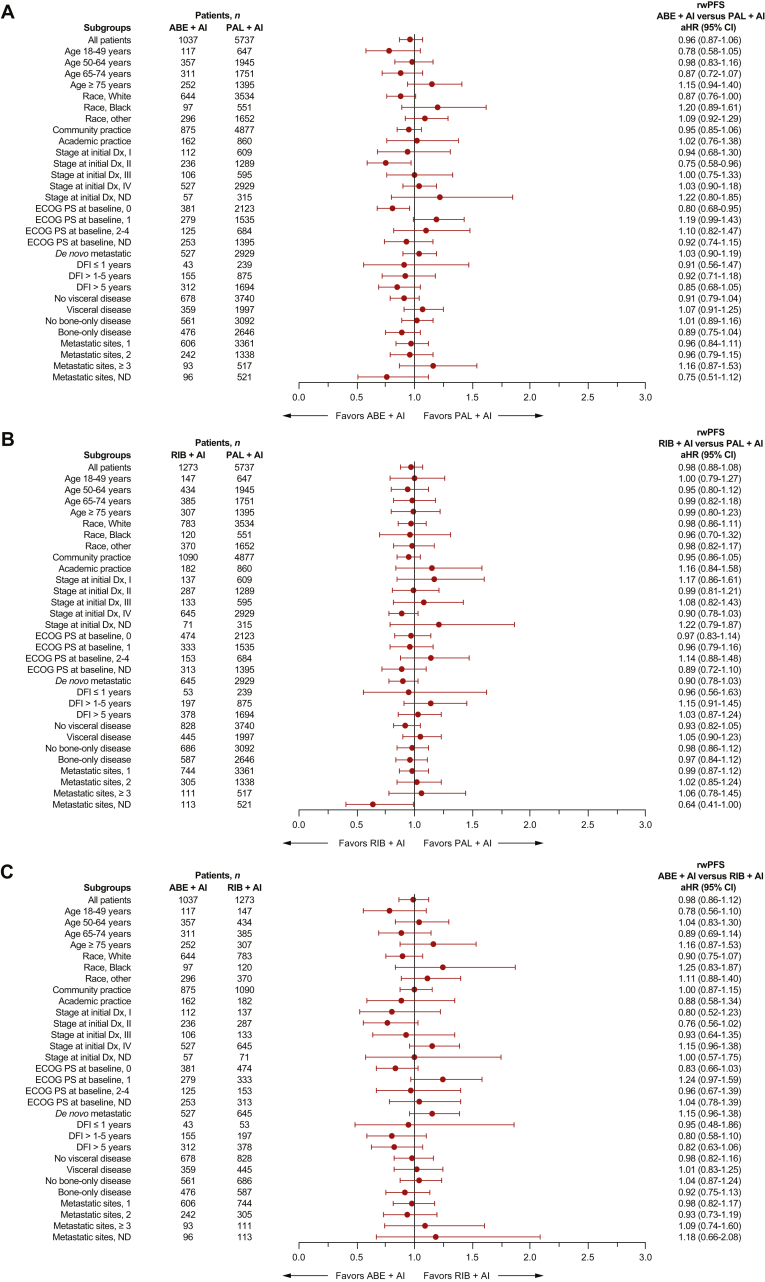
**Forest plot of rwPFS by subgroup after sIPTW in patients who started index treatment from 2017.** (A) Abemaciclib + AI versus palbociclib + AI, (B) ribociclib + AI versus palbociclib + AI, and (C) abemaciclib + AI versus ribociclib + AI. ABE, abemaciclib; AI, aromatase inhibitor; DFI, disease-free interval; Dx, diagnosis; ECOG PS, Eastern Cooperative Oncology Group performance status; ND, not determined; PAL, palbociclib; RIB, ribociclib; rwPFS, real-world progression-free survival; sIPTW, stabilized inverse probability of treatment weighting.

In this subanalysis of patients initiating index treatment from 2017 onward, no significant differences in rwPFS were observed between treatment groups before or after sIPTW (Figure 3A and B). After sIPTW, the aHR was 0.98 (95% CI 0.88-1.08, *P* = 0.6190), 0.96 (95% CI 0.87-1.06, *P* = 0.4316), and 0.98 (95% CI 0.86-1.12, *P* = 0.8110) for the ribociclib versus palbociclib, abemaciclib versus palbociclib, and abemaciclib versus ribociclib pairwise group comparisons, respectively (Figure 3B). These findings remained consistent when pairwise group comparisons of rwPFS after sIPTW were carried out across subgroups among patients initiating treatment in 2017 or later (Figure 4).

Results in the subset of patients starting treatment in 2017 or later were further supported by a sensitivity analysis using a multivariable Cox proportional hazards model. In this sensitivity analysis, pairwise group comparisons of rwPFS showed no significant differences for ribociclib versus palbociclib (aHR 0.96, 95% CI 0.87-1.05, *P* = 0.3605), abemaciclib versus palbociclib (aHR 0.98, 95% CI 0.89-1.08, *P* = 0.6447), and abemaciclib versus ribociclib (aHR 1.02, 95% CI 0.90-1.16, *P* = 0.7407).

## Discussion

Alongside data from RCTs, real-world data play an important role in informing clinical decisions by examining outcomes in the diverse patient populations seen in routine clinical practice.^37^ In this article, we presented results from P-VERIFY, the largest real-world comparative effectiveness study to date evaluating rwPFS in patients receiving CDK4/6 inhibitor plus AI treatment. The analysis found no significant differences in rwPFS (all aHR 0.96-1.02, *P* > 0.05) between 1L palbociclib, ribociclib, and abemaciclib, in combination with an AI, among patients with HR-positive/HER2-negative mBC treated in routine clinical practice in the United States.

In registrational phase III RCTs, 1L treatment with palbociclib, ribociclib, or abemaciclib plus an AI consistently demonstrated a significant prolongation of PFS compared with placebo plus an AI in patients with HR-positive/HER2-negative mBC, with a similar reduction in the risk of disease progression across trials.^8^, ^9^, ^10^ In PALOMA-2, palbociclib plus letrozole significantly improved median PFS compared with placebo plus letrozole (27.6 versus 14.5 months; HR 0.56, 95% CI 0.46-0.69, *P* < 0.0001).^8^ Similarly, median PFS was significantly prolonged in the ribociclib plus letrozole versus placebo plus letrozole cohort in MONALEESA-2 (25.3 versus 16.0 months; HR 0.57, 95% CI 0.46-0.70, *P* = 9.63 × 10^−8^) and in the abemaciclib plus AI (anastrozole or letrozole per physician’s choice) versus placebo plus AI cohort in MONARCH-3 (28.2 versus 14.8 months; HR 0.54, 95% CI 0.42-0.70, *P* = 0.000002).^9^^,^^10^ In each study, virtually all patient subgroups had a PFS benefit from the addition of a CDK4/6 inhibitor to an AI.^8^, ^9^, ^10^ In alignment with the findings of our present analysis, prior indirect treatment comparisons using data from PALOMA, MONALEESA, and MONARCH clinical trials have shown no significant differences in PFS between palbociclib, ribociclib, and abemaciclib used in combination with ET.^38^, ^39^, ^40^

Beyond indirect treatment comparisons using clinical trial data, multiple real-world studies have also compared rwPFS in patients with HR-positive/HER2-negative mBC receiving different CDK4/6 inhibitors in combination with ET.^15^, ^16^, ^17^, ^18^, ^19^, ^20^, ^21^, ^22^, ^23^, ^24^, ^25^, ^26^ To our knowledge, P-VERIFY was the largest real-world study conducted to date that evaluated the comparative effectiveness between the three approved CDK4/6 inhibitors in combination with AI. Most real-world comparative effectiveness studies to date have consistent findings with our study and have not demonstrated significant differences in rwPFS between CDK4/6 inhibitors in combination with ET.^15^, ^16^, ^17^^,^^20^^,^^22^^,^^24^, ^25^, ^26^ For example, in a prospective study of the OPAL registry in Germany (*N* = 623), there was no significant difference in rwPFS between 1L palbociclib and ribociclib in combination with ET (aHR 1.01, 95% CI 0.80-1.27).^24^ Similarly, a multicenter retrospective analysis in Turkey (*N* = 600) found no significant differences in rwPFS between palbociclib or ribociclib plus letrozole (*P* = 0.953).^20^ Notably, a few studies did not conduct tests for statistical significance when evaluating rwPFS.^18^^,^^21^

To the best of our knowledge, two real-world studies, a retrospective–prospective multicenter study in Italy (PALMARES-2)^23^ and a retrospective database analysis in Denmark,^19^ reported significant rwPFS differences between CDK4/6 inhibitor plus ET treatment groups. Both studies showed longer rwPFS in patients with HR-positive/HER2-negative mBC treated with 1L abemaciclib or ribociclib plus ET than those treated with palbociclib plus ET.^19^^,^^23^ In PALMARES-2 (*N* = 1982), aHR for rwPFS in the 1L setting was 0.83 (95% CI 0.73-0.95, *P* = 0.007) for ribociclib versus palbociclib, 0.76 (95% CI 0.63-0.92, *P* = 0.004) for abemaciclib versus palbociclib, and 0.91 (95% CI 0.73-1.14, *P* = 0.425) for abemaciclib versus ribociclib.^23^ In the 1L cohort of the Danish study (*n* = 1554), aHR was 0.80 (95% CI 0.68-0.96, *P* = 0.01) and 0.74 (95% CI 0.60-0.90, *P* = 0.005) for ribociclib or abemaciclib, respectively, versus palbociclib.^19^ The differences in rwPFS data across prior real-world studies may be due to variation in sample size, follow-up duration, baseline patient characteristics, statistical methodologies (e.g. differences in the baseline covariates that were incorporated into multivariable regression models), and/or definitions of disease progression or rwPFS, among other factors. For example, in the Danish study, rwPFS was defined as the time interval from the date of metastatic disease diagnosis, rather than 1L treatment initiation, until the date of disease progression or death in 1L.^19^ This definition can overestimate PFS, as shown by comparisons of median PFS reported in the Danish study^19^ (e.g. 32.0 months for 1L palbociclib plus ET and 42.4 months for 1L ribociclib plus ET) versus RCTs (e.g. 27.6 months for 1L palbociclib plus letrozole in PALOMA-2^8^ and 25.3 months for 1L ribociclib plus letrozole in MONALEESA-2^9^) and real-world studies (e.g. 22.7 and 22.9 months after sIPTW for 1L palbociclib plus AI and ribociclib plus AI, respectively, in the current study). Furthermore, the Danish study only adjusted for age, disease presentation, endocrine backbone, and endocrine sensitivity when comparing rwPFS between CDK4/6 inhibitors plus ET.^19^ In PALMARES-2, rwPFS was defined as the time interval between CDK4/6 inhibitor plus ET initiation and the detection of disease progression. Patients without an rwPFS event were censored at the time of data cut-off or last follow-up, if the latter occurred before data cut-off.^23^ For any given patient, if the next line of therapy was initiated due to reasons other than disease progression (e.g. toxicity), then the first instance of disease progression could have happened in the second line or later. If disease progression was not limited to the 1L setting, then rwPFS values reported in PALMARES-2 were likely overestimated.

Key strengths of our study include the diversity of patients represented and the comprehensiveness of longitudinal data collected from United States routine clinical practice. All electronic health record-derived data in the Flatiron Health database have been validated using quality and performance assessment frameworks, as described previously.^31^, ^32^, ^33^ Our study included data for a total of 9146 patients, making it the largest real-world study to date evaluating the comparative effectiveness of the three CDK4/6 inhibitors among patients with HR-positive/HER2-negative mBC. In addition, multiple baseline demographic and clinical characteristics were adjusted for using sIPTW or a multivariable Cox proportional hazards model. Finally, the robustness of our findings was evidenced by the consistency of results across different comparative methods, including sIPTW (primary analysis) and multivariable (sensitivity analysis) adjustment, and in the subanalysis of patients treated from 2017 onward (when all three CDK4/6 inhibitors were commercially available in the United States).

This study has several limitations. First, P-VERIFY was a retrospective database analysis of electronic health records, which may have inaccurate or incomplete data capture. Although sIPTW and multivariable analyses were employed to address potential bias in treatment selection, these methods cannot account for unmeasured covariates, such as prior adjuvant therapies and subsequent medications. Second, disease progression was based on treating physicians’ clinical assessments or interpretation of radiographic scans or pathology results, rather than standard criteria such as Response Evaluation Criteria in Solid Tumors. However, Flatiron Health has validated their approach to identify real-world disease progression.^36^ Furthermore, among 4235 patients who had disease progression events during follow-up, 90.9% were assessed based on imaging tests. The proportion of patients with disease progression events assessed with imaging tests was very similar among the three CDK4/6 inhibitor groups: 91.0% in palbociclib plus AI, 90.0% in ribociclib plus AI, and 91.4% in abemaciclib plus AI. Third, the ribociclib and abemaciclib groups had short follow-ups and small sample sizes compared with the palbociclib group. Fourth, the study did not have the number of progression events required for a formal, powered noninferiority design to compare treatment effectiveness. Fifth, the three CDK4/6 inhibitors have different safety profiles,^41^, ^42^, ^43^ which may have a potential impact on treatment selection, dose adjustments, duration of treatment, and disease progression. Although sIPTW was used to balance baseline patient characteristics and findings from primary analysis with sIPTW and sensitivity analyses were consistent, treatment selection bias could not be completely excluded due to unmeasured confounders, such as endocrine sensitivity and adjuvant therapies. Endocrine sensitivity and adjuvant therapies and data on safety, dose adjustments, and adherence should be considered in future research. Finally, our findings may not generalize to patient populations that are absent or underrepresented in the Flatiron Health database.

## Conclusion

P-VERIFY is the largest real-world study to date that evaluated the comparative effectiveness of the three approved CDK4/6 inhibitors. We observed no statistically significant differences in rwPFS in patients with HR-positive/HER2-negative mBC receiving 1L palbociclib, ribociclib, or abemaciclib, in combination with an AI, in routine clinical practice in the United States. Together with efficacy and safety data from RCTs and other real-world comparative effectiveness studies, these findings can help guide the selection of a CDK4/6 inhibitor for patients with HR-positive/HER2-negative mBC.
