# Supplementary material for: Real-world progression-free survival of CDK4/6 inhibitors plus an aromatase inhibitor in HR-positive/HER2-negative metastatic breast cancer in United States routine clinical practice
Source: ESMO Open. 2025 Sep 1;10(9):105570. doi: 10.1016/j.esmoop.2025.105570 (PMC12424423; doi:10.1016/j.esmoop.2025.105570)
Supplement: Supplementary Material [file mmc1.docx]

Supplementary Figure S1. Flowchart of the study cohort
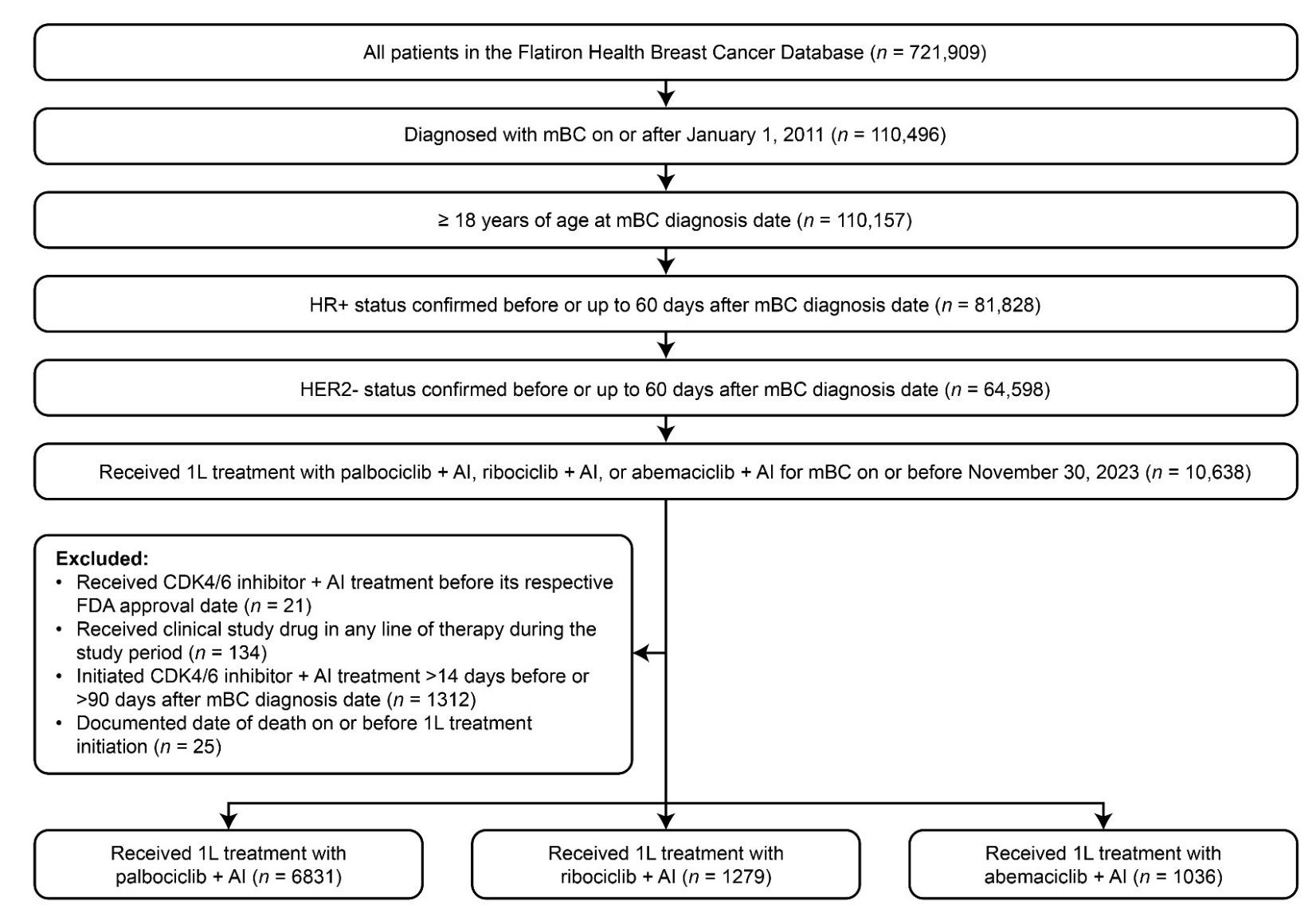


This figure is adapted from Rugo et al,^27^ licensed under [CC BY 4.0](https://s100.copyright.com/AppDispatchServlet?publisherName=ELS&contentID=S205970292401874X&orderBeanReset=true&orderSource=Phoenix).

Abbreviations: 1L, first-line; AI, aromatase inhibitor; CDK4/6, cyclin-dependent kinase 4/6; FDA, US Food and Drug Administration; HER2−, human epidermal growth factor receptor 2-negative; HR+, hormone receptor-positive; mBC, metastatic breast cancer.

Supplementary Table S1. Patient characteristics

| **Characteristic** | **Unadjusted analysis** | | | | | | **After sIPTW** | | | | | |
| --- | --- | --- | --- | --- | --- | --- | --- | --- | --- | --- | --- | --- |
|  | **Cohort** | | | **Standardized difference** | | | **Cohort** | | | **Standardized difference** | | |
|  | **PAL + AI**  **(*n* = 6831)** | **RIB + AI**  **(*n* = 1279)** | **ABE + AI**  **(*n* = 1036)** | **RIB + AI vs PAL + AI** | **ABE + AI vs PAL + AI** | **ABE + AI vs RIB + AI** | **PAL + AI**  **(*n* = 6832)** | **RIB + AI**  **(*n* = 1274)** | **ABE + AI**  **(*n* = 1038)** | **RIB + AI vs PAL + AI** | **ABE + AI vs PAL + AI** | **ABE + AI vs RIB + AI** |
| Age at mBC diagnosis, years | | | | | | |  |  |  |  |  |  |
| Mean (SD) | 65.6 (11.4) | 62.3 (12.8) | 63.3 (12.5) | -0.2725 | -0.1934 | 0.0784 | 65.0 (11.7) | 64.6 (12.0) | 64.7 (12.1) | -0.0297 | -0.0198 | 0.0097 |
| Median (IQR) | 66.0 (16.0) | 64.0 (19.0) | 64.0 (18.0) |  |  |  | 66.0 (17.0) | 66.0 (17.0) | 66.0 (17.0) |  |  |  |
| Sex, *n* (%) | | | | | | |  |  |  |  |  |  |
| Male | 75 (1.1) | 7 (0.5) | 12 (1.2) | -0.0610 | 0.0057 | 0.0665 | 70 (1.0) | 11 (0.9) | 11 (1.1) | -0.0178 | 0.0068 | 0.0245 |
| Female | 6756 (98.9) | 1272 (99.5) | 1024 (98.8) |  |  |  | 6762 (99.0) | 1263 (99.1) | 1026 (98.9) |  |  |  |
| Race, *n* (%) | | | | | | |  |  |  |  |  |  |
| White | 4385 (64.2) | 759 (59.3) | 572 (55.2) | -0.0999 | -0.1839 | -0.0836 | 4272 (62.5) | 797 (62.6) | 654 (63.0) | 0.0008 | 0.0093 | 0.0086 |
| Black | 608 (8.9) | 125 (9.8) | 124 (12.0) | 0.0300 | 0.1005 | 0.0706 | 638 (9.3) | 117 (9.2) | 94 (9.1) | -0.0063 | -0.0081 | -0.0018 |
| Other | 1838 (26.9) | 395 (30.9) | 340 (32.8) | 0.0878 | 0.1294 | 0.0415 | 1922 (28.1) | 360 (28.3) | 290 (27.9) | 0.0032 | -0.0048 | -0.0081 |
| Practice type, *n* (%) | | | | | | |  |  |  |  |  |  |
| Community | 5696 (83.4) | 1140 (89.1) | 902 (87.1) | 0.1675 | 0.1039 | -0.0639 | 5782 (84.6) | 1085 (85.2) | 872 (84.0) | 0.0154 | -0.0169 | -0.0323 |
| Academic | 1135 (16.6) | 139 (10.9) | 134 (12.9) |  |  |  | 1050 (15.4) | 189 (14.8) | 166 (16.0) |  |  |  |
| Insurance type, *n* (%) | | | | | | |  |  |  |  |  |  |
| Commercial health plan plus any other | 2515 (36.8) | 411 (32.1) | 340 (32.8) | -0.0986 | -0.0840 | 0.0146 | 2466 (36.1) | 433 (34.0) | 355 (34.2) | -0.0434 | -0.0399 | 0.0034 |
| Commercial health plan | 2143 (31.4) | 495 (38.7) | 401 (38.7) | 0.1541 | 0.1542 | 0.0001 | 2167 (31.7) | 472 (37.1) | 392 (37.8) | 0.1128 | 0.1281 | 0.0152 |
| Medicare | 323 (4.7) | 44 (3.4) | 27 (2.6) | -0.0651 | -0.1131 | -0.0487 | 310 (4.5) | 50 (3.9) | 30 (2.8) | -0.0292 | -0.0898 | -0.0610 |
| Medicaid | 116 (1.7) | 34 (2.7) | 15 (1.4) | 0.0658 | -0.0201 | -0.0854 | 121 (1.8) | 32 (2.5) | 13 (1.3) | 0.0528 | -0.0387 | -0.0907 |
| Other payer type | 1734 (25.4) | 295 (23.1) | 253 (24.4) | -0.0542 | -0.0223 | 0.0319 | 1768 (25.9) | 286 (22.4) | 248 (23.9) | -0.0809 | -0.0466 | 0.0343 |
| Disease stage at initial diagnosis, *n* (%) | | | | | | |  |  |  |  |  |  |
| I | 719 (10.5) | 126 (9.9) | 119 (11.5) | -0.0223 | 0.0307 | 0.0530 | 720 (10.5) | 136 (10.7) | 112 (10.8) | 0.0052 | 0.0091 | 0.0039 |
| II | 1552 (22.7) | 308 (24.1) | 199 (19.2) | 0.0322 | -0.0863 | -0.1185 | 1538 (22.5) | 287 (22.5) | 238 (22.9) | 0.0005 | 0.0092 | 0.0088 |
| III | 732 (10.7) | 136 (10.6) | 108 (10.4) | -0.0027 | -0.0095 | -0.0068 | 731 (10.7) | 136 (10.7) | 110 (10.6) | -0.0003 | -0.0044 | -0.0041 |
| IV | 3452 (50.5) | 638 (49.9) | 562 (54.2) | -0.0130 | 0.0744 | 0.0874 | 3473 (50.8) | 645 (50.6) | 523 (50.4) | -0.0046 | -0.0096 | -0.0050 |
| Not documented | 376 (5.5) | 71 (5.6) | 48 (4.6) | 0.0021 | -0.0397 | -0.0418 | 370 (5.4) | 70 (5.5) | 56 (5.4) | 0.0027 | -0.0023 | -0.0051 |
| ECOG PS, *n* (%) | | | | | | |  |  |  |  |  |  |
| 0 | 2363 (34.6) | 498 (38.9) | 414 (40.0) | 0.0902 | 0.1112 | 0.0210 | 2444 (35.8) | 457 (35.9) | 369 (35.5) | 0.0025 | -0.0050 | -0.0075 |
| 1 | 1809 (26.5) | 343 (26.8) | 266 (25.7) | 0.0076 | -0.0184 | -0.0260 | 1806 (26.4) | 329 (25.9) | 275 (26.5) | -0.0133 | 0.0008 | 0.0141 |
| 2, 3, or 4 | 816 (11.9) | 127 (9.9) | 100 (9.7) | -0.0646 | -0.0739 | -0.0093 | 780 (11.4) | 147 (11.5) | 119 (11.5) | 0.0032 | 0.0018 | -0.0015 |
| Not documented | 1843 (27.0) | 311 (24.3) | 256 (24.7) | -0.0610 | -0.0519 | 0.0092 | 1801 (26.4) | 341 (26.7) | 275 (26.5) | 0.0082 | 0.0035 | -0.0048 |
| Disease-free interval, *n* (%) | | | | | | |  |  |  |  |  |  |
| De novo mBC | 3452 (50.5) | 638 (49.9) | 562 (54.2) | -0.0130 | 0.0744 | 0.0874 | 3473 (50.8) | 645 (50.6) | 523 (50.4) | -0.0046 | -0.0096 | -0.0050 |
| ≤ 1 year | 265 (3.9) | 54 (4.2) | 52 (5.0) | 0.0174 | 0.0553 | 0.0380 | 276 (4.0) | 52 (4.1) | 42 (4.1) | 0.0014 | 0.0007 | -0.0007 |
| > 1-5 years | 1096 (16.0) | 195 (15.2) | 157 (15.2) | -0.0220 | -0.0245 | -0.0026 | 1082 (15.8) | 202 (15.8) | 162 (15.6) | 0.0000 | -0.0065 | -0.0065 |
| > 5 years | 2018 (29.5) | 392 (30.6) | 265 (25.6) | 0.0241 | -0.0888 | -0.1130 | 2001 (29.3) | 376 (29.5) | 311 (30.0) | 0.0044 | 0.0154 | 0.0109 |
| Visceral metastasis, *n* (%)^a^ | | | | | | |  |  |  |  |  |  |
| No | 4513 (66.1) | 839 (65.6) | 614 (59.3) | -0.0099 | -0.1409 | -0.1310 | 4458 (65.3) | 831 (65.2) | 680 (65.5) | -0.0006 | 0.0060 | 0.0066 |
| Yes | 2318 (33.9) | 440 (34.4) | 422 (40.7) |  |  |  | 2374 (34.7) | 443 (34.8) | 358 (34.5) |  |  |  |
| Bone-only metastasis, *n* (%)^b^ | | | | | | |  |  |  |  |  |  |
| No | 3608 (52.8) | 676 (52.9) | 615 (59.4) | 0.0007 | 0.1322 | 0.1314 | 3661 (53.6) | 683 (53.6) | 557 (53.7) | 0.0004 | 0.0020 | 0.0016 |
| Yes | 3223 (47.2) | 603 (47.1) | 421 (40.6) |  |  |  | 3171 (46.4) | 591 (46.4) | 481 (46.3) |  |  |  |
| Number of metastatic sites, *n* (%)^c^ | | | | | | |  |  |  |  |  |  |
| 1 | 4050 (59.3) | 752 (58.8) | 569 (54.9) | -0.0100 | -0.0883 | -0.0783 | 4010 (58.7) | 745 (58.5) | 611 (58.8) | -0.0040 | 0.0029 | 0.0069 |
| 2 | 1537 (22.5) | 310 (24.2) | 269 (26.0) | 0.0411 | 0.0809 | 0.0399 | 1585 (23.2) | 304 (23.9) | 240 (23.1) | 0.0158 | -0.0025 | -0.0183 |
| ≥ 3 | 609 (8.9) | 113 (8.8) | 105 (10.1) | -0.0028 | 0.0416 | 0.0444 | 615 (9.0) | 111 (8.7) | 92 (8.9) | -0.0112 | -0.0036 | 0.0076 |
| Not documented | 635 (9.3) | 104 (8.1) | 93 (9.0) | -0.0413 | -0.0111 | 0.0302 | 622 (9.1) | 114 (8.9) | 95 (9.2) | -0.0055 | 0.0023 | 0.0078 |
| Menopausal status at initial diagnosis, *n* (%) | | | | | | |  |  |  |  |  |  |
| Premenopausal | 1158 (17.0) | 364 (28.5) | 236 (22.8) | 0.2773 | 0.1465 | -0.1304 | 1265 (18.5) | 279 (21.9) | 204 (19.6) | 0.0849 | 0.0279 | -0.0570 |
| Postmenopausal | 5247 (76.8) | 838 (65.5) | 724 (69.9) | -0.2512 | -0.1572 | 0.0934 | 5138 (75.2) | 919 (72.1) | 762 (73.4) | -0.0702 | -0.0415 | 0.0286 |
| Not documented | 351 (5.1) | 70 (5.5) | 64 (6.2) | 0.0149 | 0.0450 | 0.0301 | 359 (5.2) | 65 (5.1) | 61 (5.9) | -0.0066 | 0.0283 | 0.0349 |
| Not applicable (patient is male) | 75 (1.1) | 7 (0.5) | 12 (1.2) | -0.0610 | 0.0057 | 0.0665 | 70 (1.0) | 11 (0.9) | 11 (1.1) | -0.0178 | 0.0068 | 0.0245 |
| Year of index date, *n* (%) | | | | | | |  |  |  |  |  |  |
| 2015 | 441 (6.5) | 0 | 0 |  |  |  | 440 (6.4) | 0 | 0 |  |  |  |
| 2016 | 655 (9.6) | 0 | 0 |  |  |  | 657 (9.6) | 0 | 0 |  |  |  |
| 2017 | 712 (10.4) | 69 (5.4) | 0 |  |  |  | 713 (10.4) | 70 (5.5) | 0 |  |  |  |
| 2018 | 789 (11.6) | 124 (9.7) | 60 (5.8) |  |  |  | 789 (11.6) | 122 (9.6) | 58 (5.6) |  |  |  |
| 2019 | 870 (12.7) | 107 (8.4) | 120 (11.6) |  |  |  | 870 (12.7) | 102 (8.0) | 125 (12.0) |  |  |  |
| 2020 | 922 (13.5) | 100 (7.8) | 147 (14.2) |  |  |  | 928 (13.6) | 92 (7.2) | 146 (14.0) |  |  |  |
| 2021 | 1027 (15.0) | 85 (6.6) | 196 (18.9) |  |  |  | 1026 (15.0) | 80 (6.3) | 189 (18.2) |  |  |  |
| 2022 | 872 (12.8) | 232 (18.1) | 239 (23.1) |  |  |  | 870 (12.7) | 226 (17.7) | 247 (23.8) |  |  |  |
| 2023 | 543 (7.9) | 562 (43.9) | 274 (26.4) |  |  |  | 539 (7.9) | 582 (45.7) | 273 (26.3) |  |  |  |
| Median follow-up duration (IQR), months | 33.0 (34.8) | 16.2 (22.5) | 21.4 (25.0) |  |  |  | 33.0 (34.7) | 15.7 (20.8) | 21.5 (25.0) |  |  |  |

This table is adapted from Rugo et al,^27^ licensed under [CC BY 4.0](https://s100.copyright.com/AppDispatchServlet?publisherName=ELS&contentID=S205970292401874X&orderBeanReset=true&orderSource=Phoenix).

^a^ Visceral disease is defined as metastatic disease in the lung and/or liver; patients could have had other sites of metastases.
^b^ Bone-only disease is defined as metastatic disease in the bone only.
^c^ Multiple metastases at the same site were counted as one site (e.g. 3 bone metastases in the spine was considered only one site).

Abbreviations: ABE, abemaciclib; AI, aromatase inhibitor; ECOG PS, Eastern Cooperative Oncology Group performance status; IQR, interquartile range; mBC, metastatic breast cancer; PAL, palbociclib; RIB, ribociclib; SD, standard deviation; sIPTW, stabilized inverse probability of treatment weighting.

Supplementary Table S2. Characteristics of patients who started index treatment from 2017 onward

| **Characteristic** | **Unadjusted analysis** | | | | | | **After sIPTW** | | | | | |
| --- | --- | --- | --- | --- | --- | --- | --- | --- | --- | --- | --- | --- |
|  | **Cohort** | | | **Standardized difference** | | | **Cohort** | | | **Standardized difference** | | |
|  | **PAL + AI**  **(*n* = 5735)** | **RIB + AI**  **(*n* = 1279)** | **ABE + AI**  **(*n* = 1036)** | **RIB + AI vs PAL + AI** | **ABE + AI vs PAL + AI** | **ABE + AI vs RIB + AI** | **PAL + AI**  **(*n* = 5737)** | **RIB + AI**  **(*n* = 1273)** | **ABE + AI**  **(*n* = 1037)** | **RIB + AI vs PAL + AI** | **ABE + AI vs PAL + AI** | **ABE + AI vs RIB + AI** |
| Age at mBC diagnosis, years | | | | | | |  |  |  |  |  |  |
| Mean (SD) | 66.0 (11.5) | 62.3 (12.8) | 63.3 (12.5) | -0.3006 | -0.2220 | 0.0784 | 65.1 (11.8) | 64.7 (12.0) | 64.8 (12.1) | -0.0324 | -0.0241 | 0.0081 |
| Median (IQR) | 67.0 (16.0) | 64.0 (19.0) | 64.0 (18.0) |  |  |  | 66.0 (16.0) | 66.0 (17.0) | 66.0 (17.0) |  |  |  |
| Sex, *n* (%) | | | | | | |  |  |  |  |  |  |
| Male | 70 (1.2) | 7 (0.5) | 12 (1.2) | -0.0720 | -0.0057 | 0.0665 | 63 (1.1) | 12 (0.9) | 12 (1.1) | -0.0149 | 0.0049 | 0.0198 |
| Female | 5665 (98.8) | 1272 (99.5) | 1024 (98.8) |  |  |  | 5674 (98.9) | 1261 (99.1) | 1025 (98.9) |  |  |  |
| Race, *n* (%) | | | | | | |  |  |  |  |  |  |
| White | 3625 (63.2) | 759 (59.3) | 572 (55.2) | -0.0794 | -0.1632 | -0.0836 | 3534 (61.6) | 783 (61.5) | 644 (62.1) | -0.0013 | 0.0093 | 0.0106 |
| Black | 528 (9.2) | 125 (9.8) | 124 (12.0) | 0.0193 | 0.0899 | 0.0706 | 551 (9.6) | 120 (9.4) | 97 (9.4) | -0.0065 | -0.0072 | -0.0007 |
| Other | 1582 (27.6) | 395 (30.9) | 340 (32.8) | 0.0726 | 0.1142 | 0.0415 | 1652 (28.8) | 370 (29.0) | 296 (28.6) | 0.0056 | -0.0053 | -0.0109 |
| Practice type, *n* (%) | | | | | | |  |  |  |  |  |  |
| Community | 4797 (83.6) | 1140 (89.1) | 902 (87.1) | 0.1606 | 0.0969 | -0.0639 | 4877 (85.0) | 1090 (85.7) | 875 (84.4) | 0.0187 | -0.0175 | -0.0362 |
| Academic | 938 (16.4) | 139 (10.9) | 134 (12.9) |  |  |  | 860 (15.0) | 182 (14.3) | 162 (15.6) |  |  |  |
| Insurance type, *n* (%) | | | | | | |  |  |  |  |  |  |
| Commercial health plan plus any other | 2135 (37.2) | 411 (32.1) | 340 (32.8) | -0.1072 | -0.0925 | 0.0146 | 2084 (36.3) | 434 (34.1) | 355 (34.2) | -0.0463 | -0.0441 | 0.0022 |
| Commercial health plan | 1864 (32.5) | 495 (38.7) | 401 (38.7) | 0.1298 | 0.1298 | 0.0001 | 1890 (32.9) | 470 (36.9) | 394 (38.0) | 0.0840 | 0.1052 | 0.0212 |
| Medicare | 264 (4.6) | 44 (3.4) | 27 (2.6) | -0.0592 | -0.1073 | -0.0487 | 250 (4.4) | 50 (3.9) | 30 (2.9) | -0.0205 | -0.0798 | -0.0595 |
| Medicaid | 106 (1.8) | 34 (2.7) | 15 (1.4) | 0.0546 | -0.0315 | -0.0854 | 112 (2.0) | 32 (2.5) | 13 (1.3) | 0.0386 | -0.0529 | -0.0908 |
| Other payer type | 1366 (23.8) | 295 (23.1) | 253 (24.4) | -0.0178 | 0.0141 | 0.0319 | 1401 (24.4) | 286 (22.5) | 245 (23.7) | -0.0460 | -0.0178 | 0.0282 |
| Disease stage at initial diagnosis, *n* (%) | | | | | | |  |  |  |  |  |  |
| I | 609 (10.6) | 126 (9.9) | 119 (11.5) | -0.0253 | 0.0277 | 0.0530 | 609 (10.6) | 137 (10.8) | 112 (10.8) | 0.0045 | 0.0065 | 0.0020 |
| II | 1301 (22.7) | 308 (24.1) | 199 (19.2) | 0.0330 | -0.0855 | -0.1185 | 1289 (22.5) | 287 (22.6) | 236 (22.7) | 0.0026 | 0.0059 | 0.0034 |
| III | 588 (10.3) | 136 (10.6) | 108 (10.4) | 0.0124 | 0.0056 | -0.0068 | 595 (10.4) | 133 (10.4) | 106 (10.2) | 0.0023 | -0.0056 | -0.0080 |
| IV | 2914 (50.8) | 638 (49.9) | 562 (54.2) | -0.0186 | 0.0689 | 0.0874 | 2929 (51.1) | 645 (50.7) | 527 (50.8) | -0.0074 | -0.0055 | 0.0019 |
| Not documented | 323 (5.6) | 71 (5.6) | 48 (4.6) | -0.0035 | -0.0453 | -0.0418 | 315 (5.5) | 71 (5.5) | 57 (5.5) | 0.0023 | -0.0002 | -0.0024 |
| ECOG PS, *n* (%) | | | | | | |  |  |  |  |  |  |
| 0 | 2070 (36.1) | 498 (38.9) | 414 (40.0) | 0.0587 | 0.0797 | 0.0210 | 2123 (37.0) | 474 (37.3) | 381 (36.7) | 0.0053 | -0.0057 | -0.0110 |
| 1 | 1543 (26.9) | 343 (26.8) | 266 (25.7) | -0.0020 | -0.0279 | -0.0260 | 1535 (26.7) | 333 (26.1) | 279 (26.9) | -0.0136 | 0.0032 | 0.0168 |
| 2, 3, or 4 | 732 (12.8) | 127 (9.9) | 100 (9.7) | -0.0894 | -0.0987 | -0.0093 | 684 (11.9) | 153 (12.0) | 125 (12.0) | 0.0028 | 0.0024 | -0.0004 |
| Not documented | 1390 (24.2) | 311 (24.3) | 256 (24.7) | 0.0018 | 0.0110 | 0.0092 | 1395 (24.3) | 313 (24.6) | 253 (24.4) | 0.0058 | 0.0013 | -0.0045 |
| Disease-free interval, *n* (%) | | | | | | |  |  |  |  |  |  |
| De novo mBC | 2914 (50.8) | 638 (49.9) | 562 (54.2) | -0.0186 | 0.0689 | 0.0874 | 2929 (51.1) | 645 (50.7) | 527 (50.8) | -0.0074 | -0.0055 | 0.0019 |
| ≤ 1 year | 230 (4.0) | 54 (4.2) | 52 (5.0) | 0.0107 | 0.0486 | 0.0380 | 239 (4.2) | 53 (4.2) | 43 (4.2) | 0.0005 | 0.0001 | -0.0005 |
| > 1-5 years | 875 (15.3) | 195 (15.2) | 157 (15.2) | -0.0003 | -0.0029 | -0.0026 | 875 (15.2) | 197 (15.4) | 155 (15.0) | 0.0055 | -0.0074 | -0.0129 |
| > 5 years | 1716 (29.9) | 392 (30.6) | 265 (25.6) | 0.0158 | -0.0971 | -0.1130 | 1694 (29.5) | 378 (29.7) | 312 (30.1) | 0.0035 | 0.0118 | 0.0083 |
| Visceral metastasis, *n* (%)^a^ | | | | | | |  |  |  |  |  |  |
| No | 3794 (66.2) | 839 (65.6) | 614 (59.3) | -0.0117 | -0.1428 | -0.1310 | 3740 (65.2) | 828 (65.1) | 678 (65.4) | -0.0027 | 0.0047 | 0.0074 |
| Yes | 1941 (33.8) | 440 (34.4) | 422 (40.7) |  |  |  | 1997 (34.8) | 445 (34.9) | 359 (34.6) |  |  |  |
| Bone-only metastasis, *n* (%)^b^ | | | | | | |  |  |  |  |  |  |
| No | 3042 (53.0) | 676 (52.9) | 615 (59.4) | -0.0038 | 0.1276 | 0.1314 | 3092 (53.9) | 686 (53.9) | 561 (54.1) | 0.0005 | 0.0048 | 0.0043 |
| Yes | 2693 (47.0) | 603 (47.1) | 421 (40.6) |  |  |  | 2646 (46.1) | 587 (46.1) | 476 (45.9) |  |  |  |
| Number of metastatic sites, *n* (%)^c^ | | | | | | |  |  |  |  |  |  |
| 1 | 3400 (59.3) | 752 (58.8) | 569 (54.9) | -0.0099 | -0.0882 | -0.0783 | 3361 (58.6) | 744 (58.5) | 606 (58.4) | -0.0026 | -0.0026 | 0.0000 |
| 2 | 1289 (22.5) | 310 (24.2) | 269 (26.0) | 0.0416 | 0.0815 | 0.0399 | 1338 (23.3) | 305 (23.9) | 242 (23.3) | 0.0148 | -0.0003 | -0.0151 |
| ≥ 3 | 512 (8.9) | 113 (8.8) | 105 (10.1) | -0.0033 | 0.0411 | 0.0444 | 517 (9.0) | 111 (8.7) | 93 (8.9) | -0.0105 | -0.0024 | 0.0081 |
| Not documented | 534 (9.3) | 104 (8.1) | 93 (9.0) | -0.0418 | -0.0116 | 0.0302 | 521 (9.1) | 113 (8.9) | 96 (9.3) | -0.0071 | 0.0073 | 0.0144 |
| Menopausal status at initial diagnosis, *n* (%) | | | | | | |  |  |  |  |  |  |
| Premenopausal | 959 (16.7) | 364 (28.5) | 236 (22.8) | 0.2835 | 0.1526 | -0.1304 | 1073 (18.7) | 277 (21.8) | 202 (19.5) | 0.0767 | 0.0205 | -0.0562 |
| Postmenopausal | 4418 (77.0) | 838 (65.5) | 724 (69.9) | -0.2566 | -0.1625 | 0.0934 | 4305 (75.0) | 919 (72.2) | 763 (73.5) | -0.0637 | -0.0344 | 0.0294 |
| Not documented | 288 (5.0) | 70 (5.5) | 64 (6.2) | 0.0202 | 0.0503 | 0.0301 | 297 (5.2) | 64 (5.0) | 60 (5.8) | -0.0056 | 0.0282 | 0.0338 |
| Not applicable (patient is male) | 70 (1.2) | 7 (0.5) | 12 (1.2) | -0.0720 | -0.0057 | 0.0665 | 63 (1.1) | 12 (0.9) | 12 (1.1) | -0.0149 | 0.0049 | 0.0198 |
| Year of index date, *n* (%) | | | | | | |  |  |  |  |  |  |
| 2017 | 712 (12.4) | 69 (5.4) | 0 |  |  |  | 718 (12.5) | 69 (5.4) | 0 |  |  |  |
| 2018 | 789 (13.8) | 124 (9.7) | 60 (5.8) |  |  |  | 793 (13.8) | 122 (9.6) | 57 (5.5) |  |  |  |
| 2019 | 870 (15.2) | 107 (8.4) | 120 (11.6) |  |  |  | 870 (15.2) | 103 (8.1) | 126 (12.1) |  |  |  |
| 2020 | 922 (16.1) | 100 (7.8) | 147 (14.2) |  |  |  | 927 (16.2) | 92 (7.3) | 146 (14.1) |  |  |  |
| 2021 | 1027 (17.9) | 85 (6.6) | 196 (18.9) |  |  |  | 1024 (17.9) | 80 (6.3) | 189 (18.2) |  |  |  |
| 2022 | 872 (15.2) | 232 (18.1) | 239 (23.1) |  |  |  | 869 (15.1) | 225 (17.6) | 245 (23.6) |  |  |  |
| 2023 | 543 (9.5) | 562 (43.9) | 274 (26.4) |  |  |  | 537 (9.4) | 582 (45.7) | 275 (26.5) |  |  |  |
| Median follow-up duration (IQR), months | 31.3 (31.0) | 16.2 (22.5) | 21.4 (25.0) |  |  |  | 31.3 (31.0) | 15.7 (20.8) | 21.4 (25.0) |  |  |  |

This table is adapted from Rugo et al,^27^ licensed under [CC BY 4.0](https://s100.copyright.com/AppDispatchServlet?publisherName=ELS&contentID=S205970292401874X&orderBeanReset=true&orderSource=Phoenix).

^a^ Visceral disease is defined as metastatic disease in the lung and/or liver; patients could have had other sites of metastases.
^b^ Bone-only disease is defined as metastatic disease in the bone only.
^c^ Multiple metastases at the same site were counted as one site (e.g. 3 bone metastases in the spine was considered only one site).

Abbreviations: ABE, abemaciclib; AI, aromatase inhibitor; ECOG PS, Eastern Cooperative Oncology Group performance status; IQR, interquartile range; mBC, metastatic breast cancer; PAL, palbociclib; RIB, ribociclib; SD, standard deviation; sIPTW, stabilized inverse probability of treatment weighting.
